# Hospitalised Smokers' and Staff Perspectives of Inpatient Smoking Cessation Interventions and Impact on Smokers' Quality of Life: An Integrative Review of the Qualitative Literature

**DOI:** 10.1155/2023/6544215

**Published:** 2023-03-03

**Authors:** Leah Epton, Shane Patman, Tracey Coventry, Caroline Bulsara

**Affiliations:** ^1^Faculty of Medicine, Nursing and Midwifery, Health Sciences & Physiotherapy, University of Notre Dame Australia, PO Box 1225, Fremantle, Australia 6959; ^2^Hollywood Private Hospital, Monash Avenue, Nedlands, Western, Australia 6009; ^3^University of Notre Dame Australia, PO Box 1225, Fremantle, Australia 6959; ^4^School of Medicine, University of Notre Dame Australia, PO Box 1225, Fremantle, Australia 6959; ^5^School of Nursing and Midwifery and Institute for Health Research, University of Notre Dame Australia, PO Box 1225, Fremantle, Australia 6959

## Abstract

**Aim:**

To identify, integrate, and appraise the evidence on hospitalised smokers' and staff perspectives of inpatient smoking cessation interventions and the impact on smokers' quality of life.

**Design:**

The integrative review method was used to present hospitalised smokers' and staff perspectives of inpatient smoking cessation interventions. *Search Method*. This integrative review consisted of a comprehensive search on smoking cessation interventions that take place during an inpatient admission to hospital for adults (> age 18 years) of the following online databases: Ovid Medline, Joanna Briggs Institute, APA PsycInfo, CINAHL, Cochrane, Google Scholar, PEDro, and Scopus. The search strategy was inclusive of peer-reviewed studies limited to the English language or translated to English. A search of grey literature and manual searching of reference lists was also conducted to identify further studies not identified in the online database search. All studies that produced any qualitative data (i.e., qualitative, mixed methods, and surveys) on inpatient-initiated smoking cessation programs were included. Outcomes of interest are included but were not limited to education, counselling, and the use of pharmacotherapy. Studies undertaken in the psychiatric, adolescent, and paediatric settings were excluded.

**Results:**

The key findings from this integrative review included positive evaluations from both patients and staff involved in inpatient smoking cessation interventions, reporting that hospitalisation was an appropriate opportunity to address smoking cessation. A number of facilitators and barriers to inpatient smoking cessation interventions included creating a supportive patient-centred environment and consideration of the cost of nicotine replacement therapy and time to deliver inpatient smoking cessation interventions. Recommendations/preferences for future inpatient smoking cessation interventions included the use of a program champion and ongoing education to demonstrate the effectiveness of the intervention, and despite the cost of nicotine replacement therapy being identified as a potential barrier, it was identified as a preference for most patients. Although quality of life was only evaluated in two studies, statistically significant improvements were identified in both.

**Conclusion:**

This qualitative integrative review provides further insight into both clinician and patient participants' perspectives on inpatient smoking cessation interventions. Overall, they are seen to produce positive benefits, and staff training appears to be an effective means for service delivery. However, insufficient time and lack of resources or expertise appear to be consistent barriers to the delivery of these services, so they should be considered when planning the implementation of an inpatient smoking cessation intervention.

## 1. Introduction

Worldwide, tobacco use is responsible for the most preventable deaths with more than 8 million deaths annually, of which many are avoidable [1]. Over 7 million of these tobacco-related deaths are associated with direct use while 1.2 million are associated with second-hand smoke exposure [1]. It has been noted that smoking and inactivity rank among the three most adaptable risk factors for chronic disease and premature death, and it is predicted that current smokers die approximately 10 years earlier than age-matched nonsmokers [2]. Smoking is also accountable for hundreds of billions of dollars of financial damage annually, which may subsequently be avoided if smoking incidence and magnitude were reduced [3]. In 2003, the World Health Organization Framework Convention on Tobacco Control (WHO FCTC) was developed in response to the global tobacco epidemic with the goal to improve public health [1].

Currently, most hospitals do not allow patients to smoke on facility grounds, and therefore, as Reid et al. [4] have noted, hospitalisation provides an excellent opportunity for the health service to identify, actively involve and engage smokers, and initiate the provision of smoking cessation treatments, support, and follow-up. Furthermore, encouragement from a health professional in the inpatient setting is a significant external prompt for a smoker to attempt quitting. Health [5] indicated that one in every 33 approaches leads to success in smoking cessation.

An evaluation by Reid et al. [4] found that combination of SCIs such as inpatient-initiated counselling and nicotine replacement therapy (NRT) with postdischarge follow-up has demonstrated significantly higher smoking cessation rates. Furthermore, the Cochrane systematic review (SR) on ISCIs by Rigotti et al. [6] found them to be effective, regardless of the patient's admitting diagnosis, or whether the admission was to an acute or rehabilitation facility. This supports the use of ISCIs as effective interventions for smoking cessation.

In the United States, The Joint Commission [7] developed evidence-based guidelines for all hospitalised inpatients which mandate that following identification of all tobacco smokers, they are offered and/or provided with evidence-based support (counselling and medication) during their admission and on discharge from hospital, and this smoking status reassessed following discharge. Smoking cessation not only increases life expectancy, decreases the risk of associated chronic diseases [8], and reduces healthcare costs; thus, ongoing investment in evidence-based interventions to assist with smoking cessation is crucial to addressing these ongoing issues. Indeed, the relatively low cost of inpatient smoking cessation interventions (ISCIs) has been shown to be cost-effective compared to the healthcare costs associated with ongoing smoking [9].

## 2. Background

Although a SR on SCIs by Rigotti et.al [6] for hospitalised patients identified their effectiveness, the SR was limited to the inclusion of randomised controlled trials (RCTs) or quasi-RCTs. Moreover, the funding mechanism of the facility and ISCI was not explicit and did not include any qualitative data reflecting participants' perspectives on the ISCIs, and the SR has yet to be updated. Ugalde et al. [10] conducted a SR on ISCI implementation strategies and their success evaluating outcomes. The authors highlighted the need for qualitative data to provide depth and understanding of the clinical and patient experience. In addition, Sharpe et al. [11] conducted their SR on barriers to the provision of ISCIs from clinicians' perspectives and, however, did not include patient perspectives or a broader range of staff perspectives. Therefore, in order to complement and build on the work of Rigotti et al. [6], Sharpe et al. [11], and Ugalde et al. [10], this qualitative integrative review (IR) of the literature focusing on staff and participants' perspectives of ISCIs will provide the reader with a broader understanding of potential contributing factors to the success or failure of ISCIs and therefore further insight in to the processes that lead to the outcome of this kind of intervention.

## 3. The Review

### 3.1. Aim

The aim of this qualitative IR was to identify, integrate, and appraise the evidence on hospitalised smokers' and staff perspectives of ISCIs and impact on smokers' quality of life (QOL) and to explore stakeholder and participant views on inpatient smoking cessation programs.

### 3.2. Design

The IR design was selected to include qualitative data as the methods involved in SRs and meta-analyses place a greater emphasis on the quality of RCTs and levels of evidence. [12] The IR methodology produces a greater understanding of the breadth and depth of the phenomenon through the inclusion of nonexperimental and experimental research [12, 13]. It also consider questions that remain unanswered by building on previous work in the area [14]. The previous SRs conducted on this topic [6, 10, 11] focused on effectiveness outcomes, implementation strategies, and some limited staff perspectives on delivering ISCIs. Therefore, through the inclusion of a wider range of staff and patient perspectives and impact on QOL for patients, this qualitative IR will provide the reader with a broader understanding of potential contributing factors to the failure/success of ISCIs by providing further insight into the processes leading to the outcome of an intervention [15]. Furthermore, the insights into the feasibility of translating an ISCI to other settings such as the private sector and funding limitations/preferences are also identified.

The methodology used for this qualitative IR was based on those described by Whittemore and Knafl [12] as the framework provided address issues such as data analysis that are specific to IRs.

### 3.3. Search Methods

A search of the following online databases was conducted from January 2011 to October 2021: Ovid Medline, Joanna Briggs Institute, APA PsycInfo, CINAHL, Cochrane, Google Scholar, and Scopus. Key search terms on the variables of interest included the following: “hospitalised/hospitalized”, “inpatient”, “patient admission”, “smokers”, “smoking”, “tobacco”, “nicotine”, “smoking cessation/prevention intervention”, “counselling/behaviour therapy”, “pharmacotherapy”, “nicotine replacement therapy”, “outcome”, and “quality of life”. A search of grey literature and manual searching of reference lists was also conducted to identify further studies not identified in the online database search. The search strategy was inclusive of peer-reviewed studies limited to the English language or translated to English. Studies that addressed inpatient-initiated smoking cessation programs and addressed the outcomes of interest were included but were not limited to education, counselling, and the use of pharmacotherapy.

### 3.4. Data Collection Method

#### 3.4.1. Types of Studies

Relevant papers were limited to any study design that included qualitative findings (i.e., qualitative, mixed methods, and survey research), and therefore, any papers producing only quantitative data (i.e., RCTs, quasiexperimental RCTs, cohort, and case series) were excluded.

#### 3.4.2. Participants

All study participants were adult patients who were current smokers at the time of their hospital admission and underwent smoking cessation support.

#### 3.4.3. Inclusion

The IR included relevant papers of any design with qualitative data on inpatient smoking cessation interventions during inpatient admission to hospital for adults (> age 18).

#### 3.4.4. Exclusion Criteria

Studies that included only quantitative data; studies undertaken in the psychiatric, adolescent, and paediatric settings; and papers not published in English were excluded.

#### 3.4.5. Search Screening and Selection Process

The EndNote referencing system (version 20, 2021; Clarivate Analytics, PA, USA) was utilised to organise records and assist with the removal of duplicate studies.

### 3.5. Search Outcome

**Figure 1:**
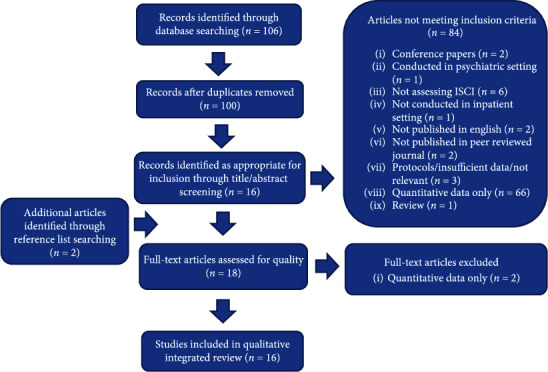
Flow diagram outlining search outcome.

The search identified 106 citations. Following the removal of duplicates, 100 studies underwent title or abstract screening with the resulting exclusion of 84 studies resulting in 16 studies. Reasons for exclusion are outlined in Figure 1. Reference lists were screened for eligible studies not previously identified, and two additional studies were included. In total, 18 full-text articles were assessed for quality.

### 3.6. Quality Appraisal

Eighteen studies were critically appraised independently by two researchers for methodological quality using standardised critical appraisal instruments. Qualitative studies were appraised using the Joanna Briggs Institute (JBI) checklist for qualitative research [16], mixed method studies were appraised using the mixed method assessment tool (MMAT) [17], and descriptive quantitative studies were appraised using the survey appraisal tool from the Center for Evidence-Based Management [18]. Following the critical appraisal process, three studies [19–21] were excluded as a result of sensitivity analyses [22].

### 3.7. Data Abstraction and Synthesis

The 16 studies that met the inclusion criteria for the qualitative IR are summarised in Table 1 under the following subheadings: design and method, sample size and location, and key findings. Outcomes from the studies included in the qualitative IR were organised, analysed and data abstracted, and synthesised using the process described by Whittemore and Knafle [12]. This process involved data reduction, data display, and data comparison facilitating the identification of “patterns, themes, variations, and relationships” [12] from which verification and conclusions can be drawn from the data collectively.

## 4. Results

The 16 studies included in the IR were conducted in Australia, Austria, Canada, China, Czech Republic, Greece, Switzerland, the United States of America (USA), and the United Kingdom (UK) and consisted of qualitative interviews (*n* = 5), mixed methods (*n* = 6), and quantitative descriptive using surveys or questionnaires (*n* = 5). Seven studies focused on patient-related outcomes, seven studies focused on staff-related outcomes, and two studies evaluated outcomes from both patients and staff involved in ISCIs. Data abstracted from the studies covered the following topics: evaluation of and attitudes towards ISCIs, frequency of provision of ISCIs, barriers to ISCIs, preferences for ISCIs, and QOL changes associated with participating in an ISCI.

The key findings from the IR included positive evaluations from patients and staff involved in ISCIs with both reporting that hospitalisation was an appropriate opportunity to address smoking cessation. A number of facilitators and barriers to ISCIs consisted of creating a supportive patient-centred environment and considering cost of NRT and time to deliver ISCIs. Recommendations/preferences for future ISCIs included the use of a program champion and ongoing education to demonstrate the effectiveness of the intervention, and despite the cost of NRT being identified as a potential barrier, it was identified as a preference for most patients. Although QOL was only evaluated in two studies, statistically significant improvements were identified in both.

### 4.1. Evaluation of and Attitudes towards ISCIs

Ten studies are reported on the evaluation of and attitudes towards ISCIs. [23–32] Overall, the evaluations and attitudes towards ISCIs were positive, and no negative comments were reported.

Patients involved in ISCIs reported that hospitalisation was an appropriately timed opportunity [28, 32] and a positive experience [24, 29] and were satisfied with the service received [30]. Finkelstein and Cha [26] assessed the feasibility of using of a mobile app for their ISCI. In their study, over 92% of the participants said they would recommend the use of the app to other hospitalised smokers.

Staff participants also reported that hospitalisation was an appropriate and effective time to approach smoking cessation [23]. Participants reported that they enjoyed providing counselling as part of the ISCI [25], and the staff found that the processes involved in ISCIs saved time [31]. In addition, the staff found that they had increased confidence in their ability to deliver smoking cessation services [27, 30] and believed that the intervention provided an important service that was helpful for patients [27, 30].

### 4.2. Provision of ISCI Services

Four studies [27, 30, 31, 36] evaluated the delivery rate of ISCI services following a training period to introduce ISCIs through the use of surveys. Despite all four studies only consisting of 1–1.5 hours of training for staff, provision of services increased by 10–29% following the training period, demonstrating that even a short period of training increased the chance of a patient receiving an ISCI.

### 4.3. Facilitators and Barriers to ISCPs

Facilitators to ISCIs were discussed by one study [34] from a nursing perspective. The authors suggested that to ensure effective ISCIs and a positive outcome, the following was essential: a patient-centred and supportive environment, encouragement of lifestyle modification, appropriately timed counselling, and onward referral as required. This involved building teams to support the patient, as per Li et al. [34] (p4788), who reported that when health professionals worked together to emphasise the advantages of smoking cessation, “they are more effective at promoting smoking cessation counselling and motivating patients to quit smoking.”

Barriers to ISCPs were discussed in five of the studies [25, 27, 32–34]. Patient-related barriers included the cost of pharmacotherapy had it not been provided as part of an ISCI [32], fear of becoming tense, experiencing mood swings, gaining weight, failing to stop smoking [38], and overall lack of interest or resistance from patients. [27, 33] Organisational barriers included lack of expertise among staff to deliver an ISCI [32, 33], shortage of coordinators who are willing to take charge of the program [25], insufficient time [27, 32–34], lack of resources [33, 34], and the presence of smoking areas on site [33].

### 4.4. Recommendations and Preferences for ISCIs

Recommendations for future ISCIs were proposed in four studies [23, 25, 27, 34]. Recommendations from patients included longer follow-up periods and improved access to smoking cessation medications [25].

Recommendations from staff perspectives consisted of the appointment of a program coordinator or champion [23, 27], ensuring resources are readily available, scheduling specific times for counselling sessions, having simple to use documentation templates for guiding the delivery of ISCIs [27], and ongoing promotion and training for staff including the demonstration of effectiveness of the program [23].

Patient preferences for ISCIs were provided by three studies [24, 37, 38]. Dobrinas et al. [24] reported on initial assessment that only 15% of their program participants were interested in receiving NRT; however, no other preferences were assessed. Interestingly, following only one to two hospital visits, one month following discharge, 20% of participants were using NRT and readiness to quit improved in 53% of patients. Thomas et al. [37] reported medication (49.5%), followed by “cold turkey” (33.5%), and gradual reduction (13.3%) as preferred strategies for smoking cessation. Within the medication preferences, NRT patches were the most popular (54.2%), then tablets (45%), inhalers (40.8%), lozenges (34.7%), e-cigarettes (32.3%), gum (27%), and sublingual tablets (23%). This is further supported by York et al. [38] who identified that not only was the use of an NRT patch considered a preferred cessation aid but also most patients were willing to pay for NRT patches on discharge as an ongoing cessation aid.

### 4.5. QOL Changes

Impact on QOL was assessed in two mixed method studies using survey-based methods [29, 35]. Politis et al. [35] found statistically significant improvements in SF-36 scores in both groups who participated in an ISCI. Schoberberger et al. [29] reported statistically significant improvements in lifestyle satisfaction using the standardised German Fragebogen zur Erfassung des Gesundheitsverhaltens (FEG) questionnaire for ex-smokers versus continuing smokers.

## 5. Discussion

The findings from this review complement the previous work in this area by Rigotti et al. [6], Sharpe et al. [11], and Ugalde et al. [10], by qualitatively presenting participants' perspectives on ISCIs including the impact on QOL for patients.

Although Rigotti et al. [6] identified that ISCIs are effective, no qualitative data or participants' perspectives were included within the review. This present qualitative IR identified that both patients' and staff experiences of ISCIs were positive experiences and an important and useful starting point to smoking cessation and therefore support the findings by Rigotti et al. [6].

The SR conducted by Sharpe et al. [11] on barriers to the provision of ISCIs from clinicians' perspectives was limited as they did not include patient perspectives or a broader range of staff perspectives. Although the barriers presented in this review were similar to those highlighted by Sharpe et al. [11], additional perspectives from both patients and staff included clinician recommendations for future ISCIs and patient preferences for ISCIs and impact on QOL.

Ugalde et al. [10], in their SR on ISCI implementation strategies and evaluation of their success, identified that brief intervention strategies alone are insufficient for long-term success with rates of delivery of ISCIs. This is reflected in the findings from this IR, which identified that staff recommended the appointment of a ISCI program coordinator or champion [23, 27] to provide ongoing support for staff. However, despite these recommendations, the findings from this qualitative IR identified that following 1-1.5 hours of staff training, delivery of ISCIs improved at two months [36] and up to 15 months [27] posttraining. Therefore, despite the findings from Ugalde et al. [10], this qualitative IR indicates that brief training is still effective for those organisations investing in the delivery of ISCIs.

A more recent study [39] on hospital staff perspectives on the provision on inpatient smoking cessation services concurred with findings from this IR that time constraints and lack of knowledge and resources are barriers to the delivery of these services. Russell et al. [39] also identified that staff believe that all members who are part of the hospital workforce should be involved in ISCIs in order to ensure the consistent delivery of the smoking cessation message. Additional barriers identified included patient groups (e.g., mental health) and context (e.g., emergency department not deemed an appropriate location to address smoking cessation).

There are a number of limitations to this qualitative IR. Firstly, the review was limited to publications from the last 10 years, English language only publications, and studies published in peer-reviewed journals. This may have reduced the number of eligible studies in this review. Timing and funding to expand the inclusion criteria may have produced more data. Another limitation of this IR involved the appraisal of literature by two reviewers and their sensitivity analyses which lead to the exclusion of three studies. However, the use of two reviewers who independently appraised each study strengthened this IR.

Overall, this qualitative IR has added a further depth of knowledge and understanding as to why ISCIs are effective by providing an insight to patient and staff perspectives on ISCIs. However, only two studies [29, 35] assessed the impact of QOL of ISCIs, and although both demonstrated statistically significant improvements, further research in this area using additional QOL outcome measures and interviews would be beneficial to add to the qualitative findings.

Potential solutions for the successful delivery of ISCIs may include ensuring all health professionals are well supported and educated to provide this service to patients by allocating sufficient time and funding for both training (initial and refresher sessions) and delivery of the service as discussed by Reid et al. [4]. Most ISCIs are facilitated by nursing and medical staff. By focusing on a multidisciplinary approach and involving other health professionals, other than nursing and medical staff, this may reduce the staff burden and ensure the consistent delivery of the smoking cessation message. If clinicians are enthusiastic and knowledgeable and have the time to discuss the importance of smoking cessation and explore strategies to deal with cravings, this may lead to better acceptance by patients and staff.

## 6. Conclusion

This qualitative IR provides further insight into both clinician and patient participants' perspectives on ISCIs. Overall, they are considered to have positive benefits, and staff training appears to be an effective means for service delivery. However, insufficient time and lack of resources or expertise appear to be consistent barriers to the delivery of these services, so they should be considered when planning the implementation of an ISCI.

## Figures and Tables

**Table 1:** Studies that met inclusion criteria for the qualitative IR.

Author	Design and method	Sample size and site	Key findings
Bains et al. [32]	Qualitative: semistructured interviews for patients and a different interview for HCPs (nursing and medical staff), on the smoking cessation service, they were offered/offer	Random sample of patients (*n* = 30, 22 service users and 8 nonservice users) and purposive sample of HCPs (*n* = 35 to represent all specialty areas) from one hospital in the UK	Most patients felt that the service was appropriately timed and a good opportunity to attempt smoking cessation. If patients had not been approached, many patients reported that they would have attempted to quit alone, although some stated pharmacotherapy costs would have been a barrier. Service delivery by a specialist advisor was favoured by patients and HCPs, largely because HCPs lacked time and expertise to intervene.

Campbell et al. [23]	Qualitative: semistructured interviews for 12 key informants from 6 hospitals that differed on OMSC program activities (identify and document smokers, advise quitting, provide medication, and offer follow-up)	Key informants (SCCs and DMs) (*n* = 12, 2 each from 6 hospitals using the OMSC) were intentionally selected in Canada	Key informants viewed the OMSC as an effective smoking cessation intervention for the hospital setting that can reduce the prevalence of smoking in the population. Using program champions; incorporating relevant performance feedback; conducting ongoing education, training, and promotion; designating a hospital-based coordinator role; and demonstrating program effectiveness emerged as important factors for sustainability and success of the OMSC.

Jones and Hamilton [28]	Qualitative: structured interviews with patients who had participated in a new stop smoking service	Patients from 4 different wards (*n* = 44) at one hospital in the UK	All participants interviewed welcomed the opportunity to access the hospital smoking cessation service. The hospital was seen as an appropriate venue, where it was easier to make a quit attempt, and there was ready access to nicotine replacement therapy (NRT) and a supportive environment. Nine of the 19 users followed up stated that they had maintained to quit, 2 successfully went “cold turkey,” 4 said they had cut down, 2 were not clear what had happened, and 2 continued to smoke as they had prior to admission.

Katz et al. [33]	Qualitative: nurses' survey and semistructured interviews conducted in a purposeful sample to collate different attitudes toward cessation counselling	Nurses who worked on internal medicine units at four academic VA hospitals in the USA completed surveys (*n* = 164) and were interviewed (*n* = 33)	Knowledge-related and attitudinal barriers included perceived lack of skills in cessation counselling and scepticism about the effectiveness of cessation guidelines in hospitalised veterans. Nurses also reported multiple behavioural and organisational barriers to guideline adherence: resistance from patients, insufficient time and resources, the presence of smoking areas on VA premises, and lack of coordination with primary care.

Li et al. [34]	Qualitative: interviews were conducted with nurses who were qualified smoking cessation counsellors to explore their perspectives of facilitators and barriers in the implementation of effective smoking cessation counselling services for inpatients	Nurse counsellors (*n* = 16) from eleven health promotion hospitals that were smoke-free and located in China	An effective smoking cessation program should be patient-centred and provide a supportive environment. Effective smoking cessation counselling also involves encouraging patients to modify their lifestyles. Time constraints and inadequate resources are barriers that inhibit the effectiveness of smoking cessation counselling programs in acute care hospitals.

Dobrinas et al. [24]	Mixed methods: questionnaire with open- and close-ended questions to evaluate the impact of a smoking cessation intervention for hospitalised patients by a clinical pharmacist previously trained for smoking cessation counselling using change in motivational stage, abstinence at follow-up, change of readiness to quit score between hospital visit and follow-up, and patients' evaluation of the program and pharmacotherapy interventions	Hospitalised smokers (*n* = 40) who received smoking cessation intervention at a hospital in Switzerland	At least 1 month after discharge, the readiness to quit of 53% of patients improved and 33% of patients declared themselves abstinent. Even though 35% of patients declared having mild to moderate withdrawal symptoms in hospital, only 15% were interested in receiving nicotine replacement therapy. Study participants evaluated the intervention positively.

Duffy et al. [25]	Mixed methods: qualitative interviews and evaluation of volunteer telephone smoking cessation counselling follow-up program implemented as part of the inpatient Tobacco Tactics intervention using reach, effectiveness, adoption, implementation, and maintenance (RE-AIM) framework. Program evaluation included number of telephone smoking cessation counselling calls, abstinence rates, intervention costs, and program feedback from volunteers and veterans	Data was collected, and interviews are conducted with inpatient Tobacco Tactics intervention participants (*n* = 131) and volunteers (*n* = 25) at a VA hospital in the USA	19% of the sample was reached 0–1 times while 81% were reached 2–4 times. Those reached more often were more likely to quit smoking. Sixty-day 24-hour point-prevalence quit rates (abstracted from volunteer documentation) were 33% for those reached 2–4 times compared to 4% of those reached 0–1 times (*p* < 0.01) (74% follow-up rate with 34 assumed to be smokers). Themes from patient interviews revealed that veterans were enthusiastic about the program and liked and appreciated the support from the volunteers. Suggestions for improvement included more phone calls over a longer period of time and better patient access to smoking cessation medications. Volunteer counsellors expressed that they felt properly prepared for being a telephone cessation counsellor and that they enjoyed counselling veterans. In terms of maintenance, the greatest organisational barriers to implementing the program were lack of space, a coordinator who can “own” the program, and restrictions on volunteers being able to document in the EMR. The reach, effectiveness, adoption, and implementation of the program were high, and while the intervention was not maintained long term, it was maintained short term.

Duffy et al. [31]	Mixed methods: survey and interviews provided an evaluation of the nurse-administered Tobacco Tactics intervention versus usual care measuring rates of receipt/delivery of services and nurses' evaluation of the intervention	Convenience sample of patients (*n* = 1528) and nursing staff (*n* = 1720) from six Michigan Trinity Health community hospitals in the USA (matched on size and number of minority patients), of which three were to receive the nurse-administered Tobacco Tactics intervention and three were to receive usual care	In the intervention sites, more patients (39.9%) in the postintervention period reported receiving handout materials compared to the preintervention period (28.4%) (*p* < 0.001), whereas there was a decrease in receipt of handout materials in the control group pre- to postintervention (30.2% pre- versus 20.5% postintervention; *p* < 0.01). Qualitative comments were very positive (“user friendly,” “streamlined,” or “saves time”), although problems with showing patients the DVD and charting in the electronic medical record were noted.

Finkelstein and Cha [26]	Mixed methods: survey and semistructured interviews were used before and after evaluations of mobile app for the hazards of smoking education delivered via touch screen tablets to hospitalised smokers using change in hazards of smoking knowledge score (KS), smoking attitudes, and stage of change. Attitudinal surveys are used to evaluate patients' acceptance of app, and their perceptions of usability, content clarity, and usefulness of the system and interviews are used to explore participants' views on app content and interface	Active smokers (*n* = 55) consecutively admitted to two medicine units at two large urban academic teaching hospitals (location not stated)	After the mobile app use, mean KS increased from 27 (3) to 31 (3) (*p* < 0.0001). Attitudinal surveys and qualitative interviews identified high acceptance of the mobile app by hospitalised smokers. Over 92% of the study participants recommended the app for use by other hospitalised smokers, and 98% of the patients were willing to use such an app in the future.

Politis et al. [35]	Mixed methods: 52-week trial of open-label, preference-based, parallel group comparing standard regimen of varenicline combined with postdischarge advanced behavioural support (group A) or one private consultation session during hospitalisation (group B) measuring abstinence rates and change in SF36 score	Hospitalised smokers self-selected at the First Pulmonology Clinic of Kavala General Hospital, Greece, to group A (*n* = 44) or group B (*n* = 57)	At week 52, 52.3% in group A and 14% in group B were still smoking abstinent. Smoking cessation improved QoL in both groups. The comparison of mean scores between baseline and week 52 showed statistically significant changes for all SF36 domains.

Schoberberger et al. [29]	Mixed methods: abstinence rates and use of standardised questionnaire by participants who completed an inpatient smoking cessation program and explore benefit participating in the program	Patients (*n* = 207) who completed the inpatient smoking cessation program in a hospital in Austria	In 12-month postprogram completion, more than 90% of ex-smokers believe that an inpatient smoking cessation therapy has a positive effect on one's health, i.e., an encouraging, supportive environment appears to assist the cessation process. 42.6% of participants (loss to F/U 23%) were identified by carbon monoxide verifications as ex-smokers. Significant changes in lifestyle satisfaction were reported by ex-smokers compared to still smokers.

Fore et al. [27]	Quantitative descriptive: two cross-sectional surveys of nurses and other staff after participating in the Tobacco Tactics training program to determine (1) factors associated with nurses' perceived confidence in and importance of delivering cessation interventions to patients and (2) whether self-reported delivery of smoking cessation services increased after training program was implemented	Survey data collected from nurses two months after participating in the one-hour Tobacco Tactics (USA) training (*n* = 145) and again 15-month posttraining (*n* = 90)	At 15-month posttraining, the vast majority (over 85%) of staff felt at least moderately, very, or extremely confident in providing smoking cessation services and felt that providing these services was important or very important. The vast majority (nearly 90%) were somewhat or extremely satisfied with the training and agreed or strongly agreed that they had a good understanding of the elements of the intervention. The most commonly cited barriers included patients not being interested and lack of time. Common suggestions for improvement included designating key personnel to perform or coordinate smoking cessation interventions, having resources readily available, planning scheduled sessions for counselling, and improving the documentation template to improve usability. Following the training, the proportion of nurses self-reporting the provision of cessation services significantly increased from preintervention to postintervention, suggesting that the Tobacco Tactics training increased nurses' likelihood of providing smoking cessation services.

Sarna et al. [36]	Quantitative descriptive: use of surveys to evaluate self-reported frequency of nursing interventions to support patients' quit efforts in their nursing practice pre/posttraining and the impact of nurses' smoking status on program outcomes	Convenience sample of nurses (*n* = 98) from the Czech Republic who attended 1 of 10 educational programs about brief smoking cessation interventions for hospitalised smokers	At 3 months, compared to baseline, significantly (*p* < 0.05), more nurses assessed patients' interest in quitting, assisted with quit attempts, and recommended the use of the quitline for cessation. Also at 3 months, nurses who smoked were less likely to ask about smoking status (odds ratio (OR) = 4.24 and 95% confidence interval (CI; 1.71, 10.53)), advise smokers to quit (OR = 3.03, 95% CI (1.24, 7.45)), and refer patients to a quitline (OR = 2.92, 95% CI (0.99, 8.63)) compared to nonsmokers.

Thomas et al. [37]	Quantitative descriptive: face-to-face interview using a structured questionnaire to identify quit experiences and preferences for a future quit attempt among smokers	Hospitalised smokers (*n* = 600) enrolled in a smoking cessation trial from inpatient wards of three Australian hospitals	Previous quit attempts: motivation to quit smoking was high, and almost two-thirds (64.3%) of participants had tried quitting at least once during the previous 12 months. Of the participants who tried quitting in the previous 12 months, 80.6% reported experiencing at least one difficulty or withdrawal symptom during their quit attempts. Of those who tried quitting in the previous 12 months, 69.9% had used at least one method (either pharmacological or nonpharmacological support) to assist their quit attempts. Motives and preferences for future quit attempt: more than half the participants (58.5%) believed that medication would assist them to quit. The most widely selected strategy to give up smoking was “quit with the help of medicines” (49.5%), followed by “cold turkey” (33.5%) and “reduce gradually” (13.3%). Nicotine patches (54.2%) were the preferred form to assist quitting, followed by tablets (45.0%), inhalers (40.8%), lozenges, (34.7%), electronic “cigarettes” (e-cigarette) (32.3%), chewing gum (27.0%), and sublingual tablets (23.0%). There is a clear need for patient education regarding evidence-based treatments, and the implications of using unproven treatments should also be explained while also considering patient preferences.

Vick et al. [30]	Quantitative descriptive: before/after survey on receipt, satisfaction and use of services for patients (reach), and staff perceptions and delivery of service (adoption/implementation)	Survey data collected from patients (*n* = 104) and staff (*n* = 81) at Jesse Brown VA Medical Centre, USA	Postintervention patients reported receipt of services 10% more, and service satisfaction was 10% higher than preintervention patients. In both before and after intervention implementation, staff felt that the VA should be doing more to assist smokers to quit and felt that providing cessation services was important. Staff confidence in their ability to provide smoking cessation services improved greatly posttraining (*p* = 0.0017) as did self-reported delivery of smoking cessation services (*p* = 0.0154). At two-month postintervention, staff survey revealed that the vast majority of staff were extremely/somewhat satisfied with the training sessions.

York et al. [38]	Quantitative descriptive: cross-sectional survey of hospitalised medical-surgical patients who smoke determine their perceived barriers to quitting and participating in a free smoking cessation support program	Current smokers (*n* = 79) in acute care medical-surgical units at a community-based hospital in the USA	Subjects' greatest fears about quitting included becoming tense/nervous, mood swings, fear of failure, and weight gain. 59.5% of subjects stated that they would be willing to call the free statewide quitline, while 29.1% stated that they were willing to participate in a web-based cessation program. The majority of subjects preferred the nicotine patch as a cessation aid and were willing to pay for the nicotine patch as a cessation aid after discharge.

Abbreviations: DMs: decision-makers; HCPs: healthcare professionals; OMSC: Ottawa Model of Smoking Cessation; QOL: quality of life; SCCs: smoking cessation coordinators; VA: veterans' affairs.

## Data Availability

The data that support the findings of this study are available from the corresponding author upon reasonable request.

## Additional Points

*Key Point Summary*. (i) Tobacco use is responsible for most preventable deaths worldwide, with more than 8 million deaths annually attributed to tobacco use; smoking kills half of its consumers, of which many are avoidable [1]. (ii) “Hospitalisation provides a unique opportunity to identify and engage smokers, initiate cessation treatments and facilitate appropriate follow-up and support” as most hospitals do not allow patients to smoke on their grounds [4] (p11). (iii) Encouragement from a health professional is a significant external prompt for a smoker to attempt quitting [5]. (iv) Previous systematic reviews conducted on this topic [6, 10, 11] have focused on effectiveness outcomes, implementation strategies and outcomes, and some staff perspectives on delivering inpatient smoking cessation interventions. (v) To date, a qualitative review that is inclusive of all participants' perspectives on inpatient smoking cessation interventions has not been conducted. Therefore, the purpose of this integrative review is to synthesise and present qualitative research findings from the last 10 years regarding staff and patients' perspectives on participating in an inpatient smoking cessation intervention, including the impact on quality of life for patients.

## References

[1] World Health Organization (2020). *Tobacco fact sheets*.

[2] Kaczynski A. T., Manske S. R., Mannell R. C., Grewal K. (2008). Smoking and physical activity: a systematic review. *American Journal of Health Behavior*.

[3] World Health Organization (2014). *Toolkit for delivering the 5A’s and 5R’s brief tobacco interventions in primary care*.

[4] Reid R. D., Mullen K. A., Slovinec D'Angelo M. E. (2010). Smoking cessation for hospitalized smokers: an evaluation of the “Ottawa model”. *Nicotine & Tobacco Research*.

[5] Alfred Health (2017). *Clinical Management of Nicotine Dependency among Patients: Version 10*.

[6] Rigotti N. A., Clair C., Munafò M. R., Stead L. F., Cochrane Tobacco Addiction Group (2012). Interventions for smoking cessation in hospitalised patients. *Cochrane Database of Systematic Reviews*.

[7] Joint Commission (2008). The Joint Commission tobacco treatment measures: overview and current status–July 2011. *Archives of Internal Medicine*.

[8] West R., Raw M., McNeill A. (2015). Health-care interventions to promote and assist tobacco cessation: a review of efficacy, effectiveness and affordability for use in national guideline development. *Addiction*.

[9] Mullen K. A., Manuel D. G., Hawken S. J. (2017). Effectiveness of a hospital-initiated smoking cessation programme: 2-year health and healthcare outcomes. *Tobacco Control*.

[10] Ugalde A., White V., Rankin N. M. (2022). How can hospitals change practice to better implement smoking cessation interventions? A systematic review. *CA: A Cancer Journal for Clinicians*.

[11] Sharpe T., Alsahlanee A., Ward K. D., Doyle F. (2018). Systematic review of clinician-reported barriers to provision of smoking cessation interventions in hospital inpatient settings. *Journal of Smoking Cessation*.

[12] Whittemore R., Knafl K. (2005). The integrative review: updated methodology. *Journal of Advanced Nursing*.

[13] de Souza M. T., da Silva M. D., de Carvalho R. (2010). Integrative review: what is it? How to do it?. *Einstein (Sao Paulo)*.

[14] Rodgers B. L., Knafl K. A. (1993). *Concept Development in Nursing: Foundations, Techniques, and Applications*.

[15] Bazeley P. (2012). Integrative analysis strategies for mixed data sources. *American Behavioral Scientist*.

[16] JBI (2020). *Critical appraisal tools*.

[17] Hong Q. N., Fàbregues S., Bartlett G. (2018). The mixed methods appraisal tool (MMAT) version 2018 for information professionals and researchers. *Education for Information*.

[18] Center for Evidence-Based Management (2019). *What is critical appraisal?*.

[19] Katz D. A., Paez M. W., Reisinger H. S. (2014). Implementation of smoking cessation guidelines in the emergency department: a qualitative study of staff perceptions. *Addiction Science & Clinical Practice*.

[20] Trout S., Ripley-Moffitt C., Meernik C., Greyber J., Goldstein A. O. (2017). Provider satisfaction with an inpatient tobacco treatment program: results from an inpatient provider survey. *International Journal of General Medicine*.

[21] Faseru B., Turner M., Casey G. (2011). Evaluation of a hospital-based tobacco treatment service: outcomes and lessons learned. *Journal of Hospital Medicine*.

[22] Thomas J., Harden A. (2008). Methods for the thematic synthesis of qualitative research in systematic reviews. *BMC Medical Research Methodology*.

[23] Campbell S., Pieters K., Mullen K. A., Reece R., Reid R. D. (2011). Examining sustainability in a hospital setting: case of smoking cessation. *Implementation Science*.

[24] Dobrinas M., Blanc A. L., Rouiller F. (2014). Clinical pharmacist's role in implementing a smoking cessation intervention in a Swiss regional hospital: an exploratory study. *International Journal of Clinical Pharmacy*.

[25] Duffy S. A., Ewing L. A., Louzon S. A., Ronis D. L., Jordan N., Harrod M. (2015). Evaluation and costs of volunteer telephone cessation follow-up counseling for veteran smokers discharged from inpatient units: a quasi-experimental, mixed methods study. *Tobacco Induced Diseases*.

[26] Finkelstein J., Cha E. M. (2016). Using a mobile app to promote smoking cessation in hospitalized patients. *JMIR mHealth and uHealth*.

[27] Fore A. M., Karvonen-Gutierrez C. A., Talsma A. N., Duffy S. A. (2014). Nurses’ delivery of the Tobacco Tactics intervention at a veterans affairs medical center. *Journal of Clinical Nursing*.

[28] Jones S. E., Hamilton S. (2013). Introducing a new stop smoking service in an acute UK hospital: a qualitative study to evaluate service user experience. *European Journal of Oncology Nursing*.

[29] Schoberberger R., Böhm G., Schroeder Y. (2015). Heavy dependent nicotine smokers - newfound lifestyle appreciation after quitting successfully. Experiences from inpatient smoking cessation therapy. *Public Health*.

[30] Vick L., Duffy S. A., Ewing L. A., RugEn K., Zak C. (2013). Implementation of an inpatient smoking cessation programme in a veterans affairs facility. *Journal of Clinical Nursing*.

[31] Duffy S. A., Ronis D. L., Ewing L. A. (2016). Implementation of the Tobacco Tactics intervention versus usual care in Trinity Health community hospitals. *Implementation Science*.

[32] Bains M., Britton J., Marsh J., Jayes L., Murray R. L. (2014). Patients’ and healthcare professionals’ views on a specialist smoking cessation service delivered in a United Kingdom hospital: a qualitative study. *Tobacco Induced Diseases*.

[33] Katz D. A., Stewart K., Paez M. (2016). “Let me get you a nicotine patch”: nurses’ perceptions of implementing smoking cessation guidelines for hospitalized veterans. *Military Medicine*.

[34] Li I. C., Lee S. Y., Chen C. Y., Jeng Y. Q., Chen Y. C. (2014). Facilitators and barriers to effective smoking cessation: counselling services for inpatients from nurse-counsellors’ perspectives--a qualitative study. *International Journal of Environmental Research and Public Health*.

[35] Politis A., Ioannidis V., Gourgoulianis K. I., Daniil Z., Hatzoglou C. (2018). Effects of varenicline therapy in combination with advanced behavioral support on smoking cessation and quality of life in inpatients with acute exacerbation of COPD, bronchial asthma, or community-acquired pneumonia: a prospective, open-label, preference-based, 52-week, follow-up trial. *Chronic Respiratory Disease*.

[36] Sarna L. P., Bialous S. A., Kraliková E. (2014). Impact of a smoking cessation educational program on nurses’ interventions. *Journal of Nursing Scholarship*.

[37] Thomas D., Abramson M. J., Bonevski B. (2015). Quitting experiences and preferences for a future quit attempt: a study among inpatient smokers. *BMJ Open*.

[38] York N. L., Kane C., Beaton K., Keown B., McMahan S. (2017). Identifying barriers to hospitalized patients’ participation in a free smoking cessation support program. *Medsurg Nursing*.

[39] Russell L., Whiffen R., Chapman L. (2021). Hospital staff perspectives on the provision of smoking cessation care: a qualitative description study. *BMJ Open*.

